# Variations in Plasma Levels of Orally Administered Ivermectin Could Hamper Its Potential Drug Repositioning: Results of a Bioequivalence Study in Mexican Population

**DOI:** 10.3390/ph18081193

**Published:** 2025-08-13

**Authors:** Ernesto de la Puente, Carlos Ramos-Mundo, Elena I. Flores-Pérez, Arely Vergara-Castañeda, Juan Pablo Reyes-Grajeda, Liz J. Medina-Reyes, María Isabel Ruiz-Olmedo, Marco A. Loza-Mejía

**Affiliations:** 1 Chemical Sciences School, Universidad La Salle-México, Benjamín Franklin 45, Mexico City 06140, Mexico; juande@lasallistas.org.mx (E.d.l.P.); ei.flores@lasallistas.org.mx (E.I.F.-P.); mruiz@lasallistas.org.mx (M.I.R.-O.); 2 Research Group on Development and Innovation in Health and Nutrition Promotion and Education, Universidad La Salle-México, Benjamín Franklin 45, Mexico City 06140, Mexico; arely.vergara@lasalle.mx; 3 Instituto Nacional de Medicina Genómica, Periférico Sur 4809, Mexico City 14610, Mexico; jreyes@inmegen.gob.mx; 4 Facultad de Química, National Autonomous University of Mexico (UNAM), Mexico City 04510, Mexico; lizmedina@quimica.unam.mx; 5 Design, Isolation, and Synthesis of Bioactive Molecules Research Group, Universidad La Salle-México, Benjamín Franklin 45, Mexico City 06140, Mexico

**Keywords:** ivermectin, bioequivalence, drug repurposing, intrasubject variations, intersubject variations, pharmacokinetics

## Abstract

**Background/Objectives**: Despite its initial promise as a treatment for COVID-19 due to its antiviral properties, controlled randomized trials have demonstrated a lack of clinical efficacy at standard dosages. Although its overall clinical benefits remain contentious, a recent meta-analysis suggests that ivermectin may lower the risk of mechanical ventilation in COVID-19 patients. This study aims to assess the bioequivalence of different formulations of orally administered ivermectin within a Mexican population. **Methods**: A randomized, controlled bioequivalence study was conducted involving healthy volunteers who received two oral formulations of ivermectin. Plasma samples were collected at predetermined intervals for pharmacokinetic analysis. **Results**: The findings indicate significant variations in plasma concentration profiles among the evaluated formulations. Elevated inter- and intrasubject variations, independent of the formulation, highlighted implications for both clinical efficacy and safety. **Conclusions**: The potential repurposing of ivermectin for COVID-19 treatment raises concerns, particularly regarding the variability in plasma levels resulting from oral administration, which may impact its effectiveness. The study underscores the importance of pharmacokinetic properties in the repurposing of ivermectin as a therapeutic agent. Given the observed discrepancies in plasma levels, careful consideration of dosing and formulation is essential for optimizing clinical outcomes in potential new applications of ivermectin.

## 1. Introduction

Drug repurposing, also known as repositioning, is a strategic approach that identifies new therapeutic uses for existing drugs—whether approved, withdrawn, or discontinued—utilizing techniques such as bioinformatics, deep learning, and network-based analysis [1,2]. It has been estimated that more than 500 approved drugs can be repurposed [3]. This pathway offers faster development timelines, lower costs, and relies on established safety profiles, making it especially attractive during public health emergencies, such as the COVID-19 pandemic [4].

**Figure 1:**
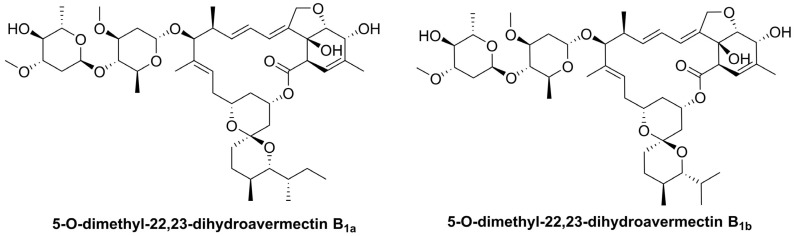
Chemical structures of ivermectin.

During this period, one of the most polemic repurposed drugs was ivermectin [5]. This broad-spectrum antiparasitic drug is a mixture of two 16-membered macrocyclic lactones named 5-*O*-dimethyl-22,23-dihydroavermectin B_1a_ and B_1b_ in a ratio of 80:20 (Figure 1). It has shown insecticidal, acaricidal, anticancer, and antiviral activities against lentivirus and flavivirus [6]. In the first stages of the pandemic, in vitro studies demonstrated its ability to inhibit SARS-CoV-2 replication within 48 h [7], by inhibiting the nuclear import of viral proteins, blocking the protease 3CLpro, and downregulating the signal transducer and activator of transcription 3 (STAT3). Though some trials suggested symptomatic improvement [8], most randomized clinical studies reported no significant efficacy at standard or moderately high doses [9,10]. Nevertheless, a recent meta-analysis reported a potential reduction in adverse outcomes, such as mechanical ventilation and adverse events [11], prompting renewed interest in ivermectin’s pharmacokinetics and reformulation strategies for repurposing [12].

In this sense, the need to develop suitable formulations that help overcome intellectual property hurdles for the new therapeutic and inclusive solution results overriding [13,14], considering that a poor pharmacokinetic profile (such as poor absorption, high plasma protein binding, and low metabolic stability) could impede the successful repurposing [15]. On this basis, pharmacokinetics of ivermectin have been extensively studied in both humans and animals [16,17], demonstrating significant pharmacokinetic variability after oral administration [18,19], which underscores the need for further studies on ivermectin’s pharmacokinetics.

Given the global demand for ivermectin during the COVID-19 pandemic, a new formulation was designed to provide a bioequivalent local alternative to the reference product. Although no specific enhancement strategy was applied to improve solubility or permeability, evaluating bioequivalence was crucial to support the interchangeable use of the product. In this work, we present the results of a bioequivalence study of an ivermectin oral formulation in the Mexican population, as well as an analysis of the factors that impact the success of studies of this type.

## 2. Results

### 2.1. Subjects

Sixty-six healthy Mexican subjects (27 males and 39 females) participated in the study, all of whom presented with normal clinical and laboratory parameters and met all other inclusion/exclusion criteria. Sixty-two subjects completed the study because one volunteer was eliminated at entry to the second period of the study for presenting a positive breathalyzer test, and three others were eliminated for non-attendance at the second period for personal reasons.

### 2.2. Pharmacokinetics

**Figure 2:**
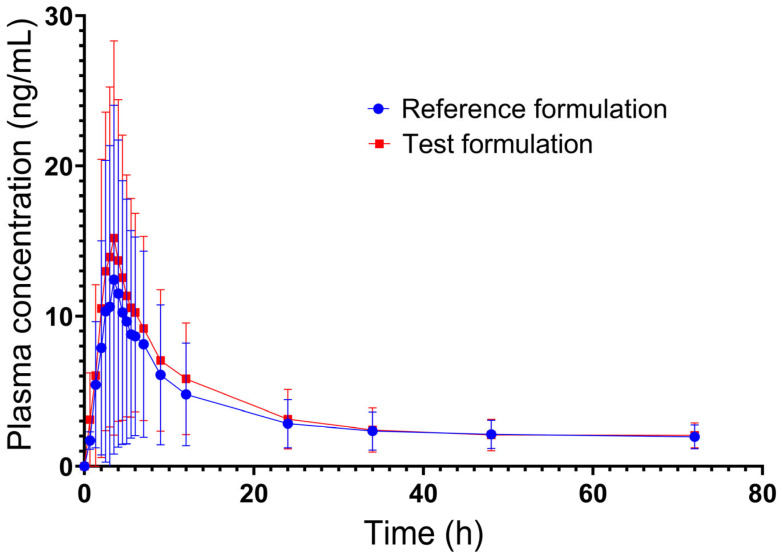
Observed ivermectin plasma concentration vs. time profiles in volunteer subjects (n = 62). Error bars show standard deviation.

The mean plasma concentration versus time curves of ivermectin are shown in Figure 2. The pharmacokinetic parameters of the test and reference products are summarized in Table 1. On average, the differences between the test and reference products for the pharmacokinetic parameters of C_max_ and AUC_0–72_ were greater than 20%. For the test formulation, the estimated t_1/2_ was approximately 22.8 h, while for the reference formulation, it was 25.1 h. Supplementary Table S1 includes the descriptive statistics of ivermectin plasma concentration data versus sampling time.

Table 2 presents the results of statistical tests applied to the log-transformed data for C_max_ and AUC_0–72_, used to determine the bioequivalence of the test and reference products at a 90% confidence level. The 90% confidence interval of the ratio of the geometric means for the test and reference products was not within the predefined equivalence range of 80% to 125% for C_max_ (109.53–148.89%) nor for AUC_0–72_ (117.48–164.79%).

Of note, the intrasubject variability for log-transformed C_max_ was 54.7% and 61.2% for AUC_0–72_. This value exceeded the 33% CV used for the study sample size calculations. Also, it is worth noting that the intersubject CV was 62.34% for C_max_ and 91.58% for AUC.

### 2.3. Safety

Adverse events were recorded for the test and reference products to assess safety. There were 38 adverse events in the study, 25 with the test drug and 13 with the reference drug, all classified as non-serious. Among the adverse events, 27 were classified as possibly related to the medication. All cases were classified as mild or moderately severe and recovered without sequelae. Six of the adverse events required medical treatment; in three of the cases, there was a headache, which was effectively resolved with Paracetamol; there was one case of abdominal pain for which butyl hyoscine was administered; there was one external case of food poisoning not associated with the medication (resolved with loratadine); and one more external case of pain in the left leg, for which treatment consisted of sulindac and diclofenac.

In summary, the test and reference formulations exhibited statistically significant differences in key pharmacokinetic parameters (C_max_ and AUC_0–72_) and failed to meet the conventional bioequivalence criteria. High inter- and intra-subject variability was observed across both formulations. These findings raise important questions regarding the clinical reliability of orally administered ivermectin, particularly in the context of drug repurposing. The following section discusses the potential mechanisms underlying these observations and their possible translational implications.

## 3. Discussion

Drug repositioning became a significant strategy during the COVID-19 pandemic due to the urgent need for rapid solutions. Innovative molecules, like nirmatrelvir in combination with ritonavir, were approved for treatment. Drug repositioning utilizes existing human safety data, allowing for quicker implementation. Research focused on in silico methods to evaluate the affinity of various molecules for SARS-CoV-2 targets, including proteases and the spike protein. Drugs that inhibit IMPα/β1-mediated nuclear import, such as ivermectin [20], were also studied. A recent meta-analysis suggested that ivermectin might reduce the risk of adverse events and mechanical ventilation in COVID-19 patients [11]. This highlights the need for well-designed clinical trials that consider factors like drug formulation, dosage, and potential interactions for future repurposing studies.

One limitation of our study is that the results showed significant differences between the test and reference ivermectin products based on pharmacokinetic parameters associated with the degree and rate of drug absorption (AUC_0–72_ and C_max_). A 72 h sampling period was established based on our national regulatory framework, which states that the sampling period should cover at least three elimination half-lives of the drug (the half-life of ivermectin reported in the prescribing information is about 18 h) and that the sampling period can be truncated at 72 h for drugs with long half-lives. Also, our choice of a 72 h sampling window follows the EMA’s immediate release (IR) product framework, which permits AUC_0–72_ as an alternative primary endpoint and does not require sampling beyond 72 h for any IR formulation, regardless of half-life. Consistent with FDA general considerations, using a truncated AUC can be appropriate for long half-life drugs when AUC extrapolation is uncertain and when the truncated window robustly captures the extent of absorption. We acknowledge that truncation can underestimate inter-period differences emerging late in the elimination phase; therefore, future studies could extend sampling to 96–120 h to enable precise estimation of AUC_0–∞_, particularly in settings with high variability. Some of these observations have been recently reviewed [21].

Additionally, the high intersubject variability (>50% for both parameters), which is even higher than what is reported in the literature (33%) and on which the sample size calculation was based [22], could potentially reduce statistical power. A replicate crossover design or the reference-scaled average bioequivalence (RSABE) approach would be necessary to estimate intrasubject variability more accurately for highly variable drugs. Future bioequivalence studies on ivermectin may benefit from employing these approaches, as recommended by FDA and EMA guidelines, to increase the statistical power and flexibility in determining equivalence. On the other hand, in the context of drug repurposing, this high variability has direct implications. Such variability increases the unpredictability of systemic exposure, which may lead to subtherapeutic plasma levels in some individuals and excessive exposure in others, thereby reducing the likelihood of achieving consistent clinical efficacy. For repurposing indications that require plasma concentrations close to the in vitro inhibitory threshold—such as proposed antiviral applications—this unpredictability poses a significant barrier to defining standardized dosing regimens. In addition, the observed variability may trigger additional regulatory scrutiny, as agencies typically require bioequivalence data with lower dispersion to support new indications.

**Figure 3:**
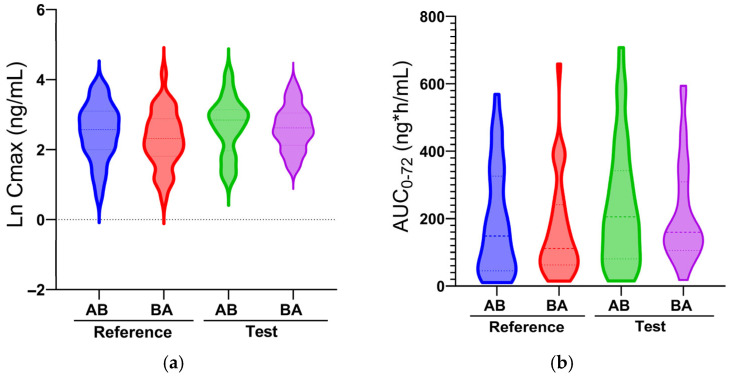
Comparison of Ln C_max_ (**a**) and AUC_0–72_ (**b**) values registered when reference (A) or test (B) formulations were administered in sequences AB or BA. The width of each violin reflects the density of individual subject values at a given parameter range, while the embedded boxplot shows the median and interquartile range.

A high intrasubject variability was also found in addition to the intersubject variability. Intrasubject variability is independent of whether the reference or test formulation was administered first, as seen in the violin plots in Figure 3, which represent the distribution of Ln C_max_ (Figure 3a) and AUC (Figure 3b) values across the four treatment arms: reference formulation administered first (AB), reference second (BA), test first (AB), and test second (BA). The shape and spread of the distributions reveal that the variability in C_max_ and AUC is similarly wide regardless of whether the reference or test formulation was administered first. Notably, the internal density patterns are comparable between the AB and BA sequences for both formulations, indicating that intrasubject variability is not driven by treatment order. This supports the assertion that the observed variability stems from intrinsic factors—such as individual absorption, metabolism, or drug transport—rather than from sequence or period effects. Furthermore, the long tails and broad spread of the violins in both formulations underscore the high intrasubject variability, consistent with the coefficients of variation (>50%) calculated elsewhere in the manuscript. Additionally, the elongated tails in both directions highlight the presence of outliers, which may contribute disproportionately to the observed coefficient of variation (>50%) and could be linked to pharmacogenetic differences or variable intestinal absorption. These plots also visually affirm the overlap in systemic exposure levels between formulations and between sequences, further justifying the lack of bioequivalence despite similar median values. Moreover, Supplementary Figure S1 presents individual subject trajectories for C_max_ and AUC_0–72_ across the two formulations. These figures that compare the individual, mean, and geometric mean of AUC and C_max_ make clear that while some subjects experienced consistent increases with the test product, others showed decreases, further confirming that variability is highly individual-specific and not strictly formulation-dependent.

**Figure 4:**
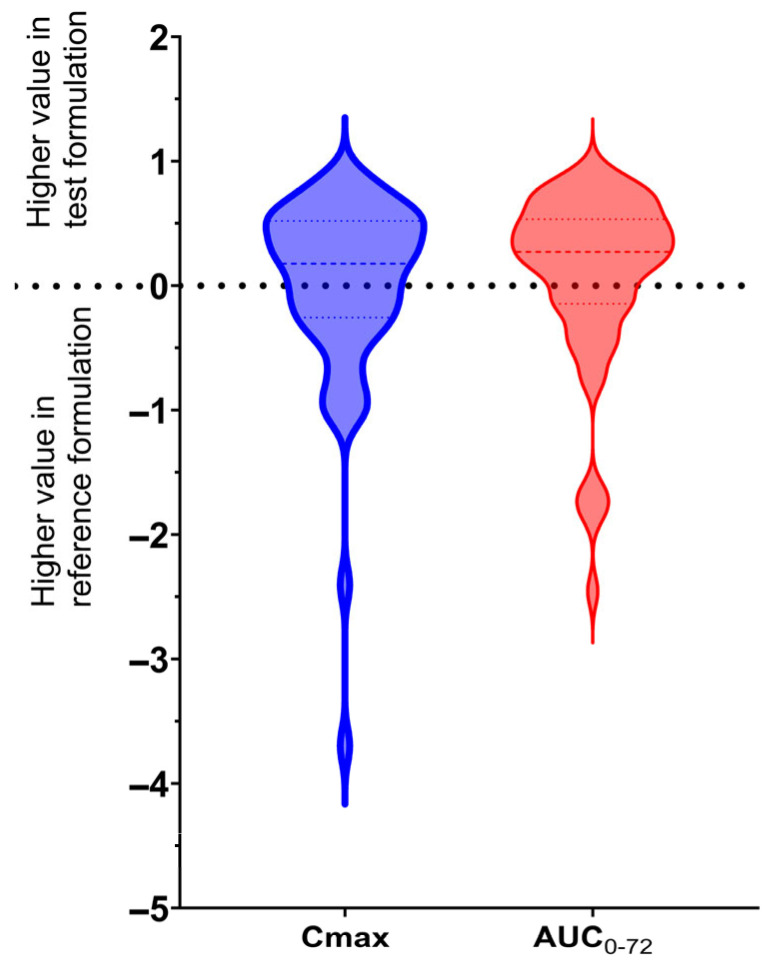
Comparison of ratio C_max_ and AUC_0–72_ between the reference and test formulation [calculated as 1 − (C_max_ in the reference formulation/C_max_ in the test formulation)]. If the ratio value is above zero, it indicates higher values in the test formulation, while negative values indicate higher values in the reference formulation. In both cases, the ratio is above zero, indicating a higher exposure to ivermectin in the test formulation.

The average maximum plasma concentration found for the reference drug in the current study conducted in a healthy adult population in Mexico was lower than those reported in populations from other geographical regions after administering a 6 mg dose of ivermectin (14.89 ± 12.07 ng/mL vs. 23.1 ng/mL) [16]. For example, Guzzo et al. reported a C_max_ of 23.1 ng/mL and a t_1/2_ of 18 ± 4 h after a 6 mg dose in U.S. volunteers [23], while Duthaler et al. reported C_max_ values of 43 ng/mL after a 12 mg dose in European healthy volunteers [18]. In contrast, our test formulation showed a C_max_ of 17.92 ± 12.99 ng/mL, and the reference formulation 14.89 ± 12.07 ng/mL, which are within the lower range but still consistent. However, T_max_, and drug exposure reached are similar to those observed in other populations like in European [24] or African [25] volunteers and patients, a fact that supports that variations in ivermectin absorption are influenced by dosage, food, metabolism, or individual characteristics [16,17]. On the other hand, Figure 4 compares the ratio of C_max_ or AUC_0–72_ values of each subject [calculated as 1 − (C_max_ in the reference formulation/C_max_ in the test formulation)]. Values above zero indicate higher exposure with the test formulation, while values below zero indicate higher exposure with the reference formulation. The plot reveals that most points lie above the zero line for both C_max_ and AUC_0–72_, suggesting a trend toward slightly greater systemic exposure with the test formulation. However, the dispersion of points is wide, with several individuals showing markedly higher or lower ratios, which underscores the high intra- and intersubject variability observed in the study. This variability persists even when a general trend is present, reinforcing the conclusion that formulation changes alone may not overcome the inherent variability of ivermectin pharmacokinetics. This behavior is characteristic of drugs with low solubility and variable intestinal absorption, such as ivermectin.

Ivermectin is metabolized by CYP3A4, and polymorphisms in this enzyme could account for variations on ivermectin’s pharmacokinetic profile. Also, it is classified as a BCS Class II drug, with low solubility and high permeability. Therefore, its oral absorption is more likely limited by solubility and influenced by P-glycoprotein (P-gp) mediated efflux [26]. Moreover, ivermectin is also a known substrate of P-gp, which is encoded by the ABCB1 gene. Since P-gp potentially limits ivermectin absorption in the gut, variability in P-gp expression, whether due to genetic polymorphisms, disease states, or drug–drug interactions, can significantly impact the pharmacokinetics of ivermectin and may explain some of the observed interindividual differences. Genetic variations in CYP3A4 and ABCB1 have been shown to significantly alter drug exposure across diverse therapeutic contexts. For example, carriers of the CYP3A4*22 allele exhibit higher tacrolimus concentrations and require lower doses to achieve target trough levels [27]. Similarly, the CYP3A4*1G variant has been associated with reduced systemic exposure to lenvatinib in Japanese thyroid cancer patients [28]. On the transporter side, ABCB1 polymorphisms also impact drug disposition. For instance, Mongolian-descendant patients with ABCB1 1236 TT or 2482-2236 CC genotypes demonstrate higher rivaroxaban C_max_ adjusted for dose and an increased bleeding risk [29]. Since, some studies have reported significant genetic variability in the *CYP3A4* [30] and *ABCB1* [31] genes in the Mexican population, which could explain important variability in drug response. Indeed, it would be important to investigate the polymorphisms in these genes and their influence on ivermectin pharmacokinetics for future studies.

Additionally, employing pharmaceutical formulations with P-gp inhibitory effects could enhance the oral bioavailability of ivermectin, as it has been demonstrated for other drugs [32]. For example, several advanced oral formulations have demonstrated the ability to enhance the bioavailability of P-gp substrates. Strategies include lipid-based systems like a self-emulsifying drug delivery system (SMEDDS), micelles, emulsions, liposomes, polymeric nanoparticles, dendrimers, and solid dispersions—all leveraging either P-gp inhibition or efflux evasion mechanisms [33,34]. Nanotechnological approaches have also been highlighted for their efficiency in bypassing intestinal efflux in oral chemotherapy models [35]. These approaches should be considered for formulations of ivermectin.

Three main observations can be made from the results of this bioequivalence study. The first consideration is the importance of taking into account the fasted–fed influence when administering ivermectin. Although this study was conducted under fasting conditions in accordance with guidance for bioequivalence trials, future studies should include a fed-state arm to better represent real-world conditions, especially given the enhanced bioavailability of ivermectin when administered with food. In general, considerations for bioavailability and bioequivalence studies indicate that drugs should be administered under a fasted state with 240 mL of water and after a 10 h fasting period to avoid the influence of GI fluid volume, pH, or gastric emptying rate. However, in their product-specific guidance, both EMA and FDA have recommendations on the study design, including requirements for food intake [36]. At least one clinical trial with ivermectin during the COVID-19 pandemic reported that the drug was administered on an empty stomach [9]. In contrast, a recent population pharmacokinetic model of ivermectin in filariasis has emphasized the convenience of administering ivermectin with food [37]. Our bioequivalence study considered fasted-state conditions, but evaluating the convenience of conducting bioequivalence studies under fed conditions would be important, particularly in designing practical therapeutic guidelines, since there were reports about the effect of food in ivermectin’s absorption [19,23,25]. Additionally, developing formulations that overcome fasted–fed variability, including osmotic delivery systems [38], amorphous solid dispersions [39], nanocrystal technologies [40], or lipid-based systems [41], is an important research area to explore [42].

In conjunction with the latter, a second observation is that formulation is crucial for drug repurposing. For example, although there is no statistically significant difference compared to the reference formulation, the test formulation achieved slightly higher levels of ivermectin. While this is not the intended objective, it highlights the importance of reformulation, as the new use of the drug may necessitate different dosage forms of formulation [43,44]. Although the test formulation did not achieve bioequivalence, its slightly higher systemic exposure and acceptable safety profile indicate potential for reformulation or optimization, particularly if considered in a fed-state scenario. These findings support the importance of formulation-specific studies in the context of drug repurposing, since variable exposure can lead to subtherapeutic levels in some patients, compromising clinical efficacy. This is especially relevant in off-label or high-dose contexts, where achieving target tissue concentrations is critical.

One final observation is the consideration of the concentration of ivermectin that would be present in the target tissues is critical. One of the main criticisms of ivermectin as a coadjuvant in COVID-19 is its relatively low potency. The in vitro evaluation of ivermectin activity against SARS-CoV-2 by Caly et al. [7] established an IC_50_ value of 1750 ng/mL. Taking into consideration previous studies of ivermectin pharmacokinetics published by Guzzo and collaborators [23] and the values of our study, it would be expected that at the maximum dose for antiparasitic effect approved by the FDA of 200 μg/kg, the adjusted C_max_ values expected at that dose, 56.48 ng/mL would be reached in the population studied by Guzzo and 29.78–35.84 ng/mL in the Mexican population, which is far from the IC_50_ value established by Caly.

However, Guzzo and collaborators established that the plasma concentration of ivermectin rises 2.5 times when administered with food. Considering this, the adjusted C_max_ value when administered with food would reach 141.21 ng/mL in Guzzo’s studied population and 74.45–89.60 ng/mL in Mexican subjects, using the values from our study. Using data from an experimental model in cattle [45], a pharmacokinetic model was built that estimated that the lung-to-plasma ratio of ivermectin would be 2.67:1. Therefore, we speculate that the administration of highest approved dose of ivermectin (200 μg/kg) for antiparasitic use, the drug concentration in lung would be C_lung_ = 377.04 ng/mL in the Guzzo’s study volunteers and C_lung_ = 198.78–239.23 ng/mL in Mexican subjects, meaning that it would require a dose 4.64 times higher (928 μg/kg) than the maximum approved dose to achieve the IC_50_ established by Caly. This value is close to the dose of 800 μg/kg used in some studies in African countries [46], which reported only non-serious adverse effects. Moreover, the study by Guzzo and collaborators reported that a single dose of 2000 μg/kg is safe [23], which is ten times higher than the maximum dose approved by the FDA. We recognize that the extrapolation of lung concentrations based on plasma values assumes linearity in distribution, which may not hold at higher doses. Nonlinear absorption kinetics and saturable tissue penetration may alter drug disposition, particularly at supra-therapeutic levels. Therefore, while the model provides a useful approximation, these values should be interpreted cautiously.

However, given the lower values calculated for C_max_, the high standard deviation, and the high CV of ivermectin, the scenario in Mexican patients would result in an estimated C_lung_ of 1987.82–2392.32 ng/mL at the maximum studied dose of 2000 μg/kg. Although this would be sufficient to achieve the IC_50_ values reported by Caly et al., the high variability of ivermectin in the Mexican population would result in only 56–64.44% of patients having the necessary ivermectin levels in their lungs. Instead, 87.29% of the volunteers of Guzzo’s study would achieve at least 1750 ng/mL. Therefore, this justifies performing bioequivalence studies under other conditions (with food intake) and observing the effect on the standard deviation and C_max_ in both plasma and lung if future repurposing studies on ivermectin are conducted. Although several factors can influence clinical outcomes, the high variability observed may increase the risk of subtherapeutic exposure in certain individuals, especially at standard doses. This could compromise efficacy in off-label uses requiring higher systemic levels, such as antiviral indications, and underscores the need for dose optimization and careful therapeutic monitoring in repurposing contexts. Therefore, future research should consider additional experimental designs, conduct pharmacogenetic analyses of CYP3A4 and ABCB1 polymorphisms and their effects on ivermectin levels and explore formulation strategies to improve its bioavailability.

## 4. Materials and Methods

### 4.1. Study Ethics

The study adhered to the Study Protocol (Protocol number CE-PEC.2043) approved by the Research Ethics Committee of the Centro Especializado en Diabetes, Obesidad y Prevención de Enfermedades Cardiovasculares (CEDOPEC, date of approval 12 May 2021) and the Federal Commission for the Protection against Sanitary Risk (COFEPRIS, number 213301410B0086/2021), the ethical principles of the Declaration of Helsinki, the International Conference on Harmonization of Good Clinical Practices guidelines, and the Mexican NOM 177-SSA1-2013, which establishes the requirements for bioequivalence studies.

### 4.2. Study Drugs

In this study, the reference product Ivexterm^®^ tablets with 6 mg ivermectin (batch no. 348445, expiry date: June 2022, manufactured in Mexico by Tecnofarma S.A. de C.V. for Grossman Laboratories S.A., Mexico City, Mexico) and the tested product Ivermectin tablets with 6 mg (batch no. 007F006, expiry date: April 2023, DLP Pharmaceutical Mexico S.A. de C.V., Mexico City, Mexico) were used. The objective was to introduce a new product that would offer a locally produced therapeutic equivalent amid high demand during the COVID-19 crisis.

### 4.3. Study Subjects

Sixty-six subjects of both sexes, from the metropolitan area of Mexico City, aged 18 to 55 years, with a body mass index (BMI) between 18 and 27 kg/m^2^, declared clinically healthy with no clinically relevant abnormalities according to their medical history, physical examination, determination of vital signs, and electrocardiogram and laboratory tests, participated in the study. The exclusion criteria included clinical evidence or disease history of hematological, renal, endocrine, pulmonary, gastrointestinal, cardiovascular, hepatic, psychiatric, or neurological disease or severe allergic reactions, hypersensitivity to the study drug or its derivatives, positive pregnancy test, positive urine test for drugs of abuse, history of drug use and/or alcoholism, and use of tobacco, alcohol, or any medication before the start and during the study. Table 3 summarizes the demographic characteristics of the study volunteers. All subjects signed the Informed Consent Form prior to the commencement of the study.

### 4.4. Study Design

The study was conducted at the Center for Scientific and Clinical Studies, S.A. de C.V. (CECyC Pharma, Santa Fe, Morelos, Mexico) in Mexico. It was carried out in 66 healthy Mexican subjects (56 + 10 reserves) of both sexes under a controlled, open-label, randomized, single-center, prospective, crossover, single-dose design, with two sequences, two periods, and two treatments staggered in two blocks, with a sampling time truncated at 72 h and a washout period of 28 days between each dose, under fasting conditions. Sample size was calculated based on a presumed intra-subject coefficient of variation of 33% for C_max_ [22], targeting 85% power and α = 0.05 under a standard two one-sided test TOST procedure. This yielded a required sample of 56 participants, with 10 additional participants reserved to account for potential dropouts. Each subject received a single oral dose of 6 mg ivermectin (equivalent to one tablet) per study period.

During the pre-selection stage of volunteers, medical history, physical examination, and laboratory and imaging studies were conducted to assess their eligibility to participate in the study. Volunteers who met the inclusion criteria and did not present any exclusion criteria were admitted to the Clinical Unit of the CECyC Pharma, 12 h before the administration, where they were tested for drug abuse and alcohol use, and, for the women volunteers, underwent a pregnancy test to verify negative results. A standardized dinner was provided for the volunteers to complete a 10 h fast before drug administration. Before the administration, a blood glucose test was conducted as part of the safety tests. Subjects received a single oral dose of 6 mg of ivermectin from either the reference formulation (treatment A) or the tested formulation (treatment B). The subjects were randomly assigned to one of two treatment sequences: sequence AB or sequence BA, receiving treatment A or B in the first dosing period and the other treatment (B or A) in the second dosing period.

Based on the half-life of ivermectin reported in the prescribing information (18 h), it was decided that sampling would be truncated at 72 h, with a washout period of at least 28 days. Blood samples were collected in vacuum tubes containing sodium citrate as an anticoagulant at the following times: 0.0 (pre-dose) and 0.67, 1.33, 2.00, 2.50, 3.00, 3.50, 4.00, 4.50, 5.00, 5.50, 6.00, 7.00, 9.00, 12.00, 24.00, 34.00, 48.00, and 72.00 h after drug administration. Subjects remained at the study center for approximately 24 h after drug administration. Samples were collected at 34.00, 48.00, and 72.00 h on an outpatient basis. Vital signs of each subject were taken before administration and on six other occasions during the study (6.00, 12.00, 24.00, 34.00, 48.00, and 72 h). During the study, the occurrence of any symptoms that might suggest the presence of adverse events (AEs) was monitored and recorded in the corresponding format.

### 4.5. Analysis Conditions

Quantification of ivermectin in plasma was performed using an analytical method with MS/MS detection coupled to a UPLC system. The chromatographic separation was performed on a Symmetry C18 column (2.1 × 50 mm, 3.5 μm). The mobile phase consisted of 20 mM ammonium acetate and 0.1% ammonium hydroxide in methanol (5:95, *v*/*v*). The flow rate was 0.2 mL/min. The compounds were detected in negative electrospray ionization with the multiple reaction monitor (MRM) mode. The MRM transitions were *m*/*z* 892.85 > 307.31 for ivermectin and *m*/*z* 640.58 > 498.36 for moxidextrin (internal standard).

Ivermectin and moxidextrin were extracted using a liquid–liquid extraction technique with methyl tert-butyl ether as the extracting agent.

Because of the sensitivity required for the characterization of the pharmacokinetic profile of ivermectin, the analytical method was validated in the range of 1.0 to 75.0 ng/mL according to the acceptance criteria defined in the Mexican Standard NOM-177-SSA1-2013 [47] for system suitability, linearity, carryover effect, sample stability, precision and accuracy within- and between-run, matrix effect, selectivity, anticoagulant effect, dilution effect, tolerance, and stability solutions.

The relationship between the chromatographic response concerning concentration in each calibration curve was fitted by linear least-squares regression to the equation y = mx + b, with arrangement 1/x^2^, where the “y” variable was the ratio of the areas of ivermectin/moxidectin obtained for the respective nominal concentration “x” of ivermectin.

The performance of the analytical run was evaluated using values obtained from the calibration and results of quality control samples. Each subject sample was integrated and quantified with the calibration curve on the day of the analysis; these curves met the linearity and accuracy parameters established during validation. Acceptance criteria for assay accuracy required intraday, interday, and reproducibility of the reinjection percentage of absorption deviation to be ≤15%, except for the lower limit of quantification, which was required to be ≤20%. Acceptance criteria for reproducibility required the interday coefficient of variation (CV) to be ≤15%, except for the lower limit of quantification, which was required to be ≤20%. The acceptance criteria for baseline selectivity required that the analytical response of interferences close to the retention time be ≤20% for the analyte of interest and ≤5% for the internal standard.

Subject samples were stored frozen at −70 °C ± 10 °C. Acceptance criteria for sample stability required that the percentage of absolute deviation was ≤15% relative to the value of recently prepared samples. Data showed that samples were stable for three freeze–thaw cycles at −70 °C; samples were also stable at room temperature and refrigeration for 23 h. Finally, the samples remained stable for at least 116 days when stored at −70 °C under freezing conditions.

### 4.6. Pharmacokinetics and Bioequivalence Analysis

Pharmacokinetic analysis, analysis of variance, and calculation of 90% confidence intervals were performed using Phoenix/WinNonlin Professional (version 8.3, Certara L.P., Princeton, NJ, USA).

Pharmacokinetic parameters were determined using noncompartmental methods and were summarized by treatment using descriptive statistics, including arithmetic mean, geometric mean, standard deviation, standard error, median, minimum, maximum, and CV for each variable. These parameters included C_max_, AUC from time zero to 72 h (AUC_0–72_), and T_max_. Natural log comparisons of the pharmacokinetic parameters C_max_ and AUC were performed for the test versus the reference drug using an analysis of variance model that included sequence, period, and formulation as fixed effects, based on linear mixed-effects models. Shapiro–Wilk’s and Levene’s tests were performed to verify normality and homogeneity of variance. Analysis of variance comparisons was performed considering the type III sum of squares. The 90% confidence intervals (CIs) of the test/reference geometric mean ratios (GMRs) for these parameters were then determined based on logarithmically transformed data. Bioequivalence between the test and reference formulations of ivermectin was demonstrated if the 90% CIs for Ln C_max_ and AUC_0–72_ were within the acceptable range of 80–125%.

## 5. Conclusions

In this bioequivalence study of two oral ivermectin formulations in healthy Mexican volunteers, high intra- and intersubject variability was observed for both C_max_ and AUC, exceeding 50% in several cases. While the test formulation tended to produce slightly higher systemic exposure than the reference, the variability was independent of treatment sequence and may limit predictable clinical performance. These findings underscore the need to consider formulation optimization, genetic variability, and dosing conditions when designing future bioequivalence and drug repurposing studies. Incorporating fed-state conditions, extended sampling to accurately estimate AUC_0–∞_, the development of more appropriate formulations, and pharmacogenetic characterization may provide a more complete understanding of ivermectin pharmacokinetics and support its safe and effective use in new therapeutic indications.

## Figures and Tables

**Table 1:** Descriptive statistics of the main pharmacokinetic parameters C_max_, AUC_0–72_, and T_max_ for reference and test formulation.

	Reference			Test		
	C_max_ (ng/mL)	AUC_0–72_ (ng/mL·h)	T_max_ (h)	C_max_ (ng/mL)	AUC_0–72_ (ng/mL·h)	T_max_ (h)
Mean	14.89	181.80	3.97	17.92	226.62	3.82
Standard deviation	12.07	152.95	1.24	12.99	166.57	1.31
Minimum	1.88	10.24	2.00	3.19	15.11	2.00
Median	11.28	134.90	3.50	14.88	167.90	3.50
Maximum	64.56	658.97	7.00	61.90	707.52	9.00
CV (%)	81.10	84.10	31.30	72.50	73.50	34.40
Geometric mean	11.02	119.23	3.80	14.07	165.89	3.63

**Table 2:** Confidence interval data. Schuirmann’s one-sided double *t*-test for determining bioequivalence (BEq).

Parameter	Ratio (%)	Classic Confidence Interval (CI)	BEq Criteria	Schuirmann’s One-Sided Double *t*-Test	BEq Criteria	TOST ^1^ Potency
Ln C_max_	127.70	(109.53–148.89)	(80.00–125.00%)	*p* (θ < 80%) = 0.00 *p* (θ < 125%) = 0.59	*p* < 0.05	0.03
Ln AUC_0–72_	139.14	(117.48–164.79)	(80.00–125.00%)	*p* (θ < 80%) = 0.00 *p* (θ < 125%) = 0.85	*p* < 0.05	<0.01

^1^ Two one-sided tests.

**Table 3:** Demographic characteristics of the studied population.

Demographic Characteristic	Mean	Std. Dev.	CV%	Range
Age (years)	29.98	9.56	31.88	18–52
Weight (kg)	62.79	10.10	16.08	41.3–88.4
Height (cm)	162	0.09	5.7	146–186
Body-mass index (BMI, kg/m^2^)	23.75	2.41	10.14	18.15–26.99

## Data Availability

Data presented in this study is contained within the article and Supplementary Materials. Further inquiries can be directed to the corresponding author.

## Supplementary Materials

The following supporting information can be downloaded at: https://www.mdpi.com/article/10.3390/ph18081193/s1, Figure S1: Comparison of individual, mean, and geometric mean of AUC and C_max_ by treatment; Table S1: Descriptive statistics of Ivermectin plasma concentration data versus sampling time.
