# Supplementary material for: Variations in Plasma Levels of Orally Administered Ivermectin Could Hamper Its Potential Drug Repositioning: Results of a Bioequivalence Study in Mexican Population
Source: Pharmaceuticals (Basel). 2025 Aug 13;18(8):1193. doi: 10.3390/ph18081193 (PMC12389682; doi:10.3390/ph18081193)
Supplement: Supplementary file 1 [file pharmaceuticals-18-01193-s001.zip › pharmaceuticals-3725613_Table S1.pdf]

A. Descriptive statistics of Ivermectin plasma concentration data versus sampling time for the reference drug N = 62.							
Time (h)	Mean (ng/mL)	Standard deviation (ng/mL)	Standard error (ng/mL)	Minimum (ng/mL)	Median (ng/mL)	Maximum(ng/mL)	% CV
0	0.00	0.00	0.00	0.00	0.00	0.00	NE
0.67	1.72	0.58	0.21	1.11	1.53	2.80	33.96
1.33	5.44	4.20	0.70	1.09	4.40	20.58	77.08
2	7.88	7.12	0.98	1.01	6.42	34.72	90.36
2.5	10.32	10.04	1.32	1.06	7.27	41.19	97.28
3	10.62	10.74	1.36	1.03	6.68	43.76	101.10
3.5	12.42	11.61	1.47	1.48	8.18	64.56	93.46
4	11.50	10.22	1.30	1.26	7.76	52.10	88.83
4.5	10.24	8.78	1.11	1.53	7.82	49.57	85.72
5	9.64	8.15	1.03	1.11	6.92	39.53	84.46
5.5	8.79	6.92	0.88	1.17	6.90	35.63	78.69
6	8.65	6.61	0.84	1.16	6.59	31.34	76.41
7	8.13	6.20	0.79	1.11	6.65	25.12	76.29
9	6.09	4.65	0.59	1.00	4.68	19.98	76.31
12	4.79	3.40	0.44	1.10	3.84	17.28	71.16
24	2.84	1.61	0.22	1.03	2.37	7.54	56.82
34	2.35	1.27	0.20	1.03	1.83	5.94	53.96
48	2.13	0.94	0.16	1.09	1.85	4.50	44.03
72	1.96	0.79	0.17	1.08	1.84	3.81	40.18

B. Descriptive statistics of Ivermectin plasma concentration data versus sampling time for the test drug N = 62.							
Time (h)	Mean (ng/mL)	Standard deviation (ng/mL)	Standard error (ng/mL)	Minimum (ng/mL)	Median (ng/mL)	Maximum(ng/mL)	% CV
0	0.00	0.00	0.00	0.00	0.00	0.00	NE
0.67	3.11	3.11	0.86	1.13	1.90	11.98	100.08
1.33	6.04	6.05	0.89	1.03	3.66	30.38	100.03
2	10.51	9.93	1.33	1.00	8.80	46.65	94.51
2.5	12.98	10.61	1.41	1.40	10.03	44.70	81.77
3	13.94	11.32	1.46	1.22	11.29	50.90	81.24
3.5	15.19	13.12	1.67	1.62	12.13	61.90	86.37
4	13.70	10.71	1.36	1.52	10.76	49.00	78.15
4.5	12.56	9.49	1.21	1.97	9.22	40.58	75.62
5	11.34	8.05	1.02	1.66	8.92	37.99	70.99
5.5	10.55	7.28	0.93	1.66	8.52	29.98	69.01
6	10.24	6.61	0.84	1.71	8.23	26.99	64.56
7	9.18	6.14	0.78	1.18	7.39	25.29	66.90
9	7.05	4.71	0.60	1.08	5.61	19.54	66.81
12	5.83	3.72	0.48	1.03	4.16	15.78	63.86
24	3.14	1.98	0.26	1.01	2.37	8.94	62.99
34	2.42	1.47	0.21	1.04	1.78	7.02	60.75
48	2.09	1.05	0.16	1.02	1.78	4.35	50.26
72	2.06	0.83	0.16	1.00	1.94	3.74	40.38
