# Supplementary figures and images for: Variations in Plasma Levels of Orally Administered Ivermectin Could Hamper Its Potential Drug Repositioning: Results of a Bioequivalence Study in Mexican Population
Source: Pharmaceuticals (Basel). 2025 Aug 13;18(8):1193. doi: 10.3390/ph18081193 (PMC12389682; doi:10.3390/ph18081193)

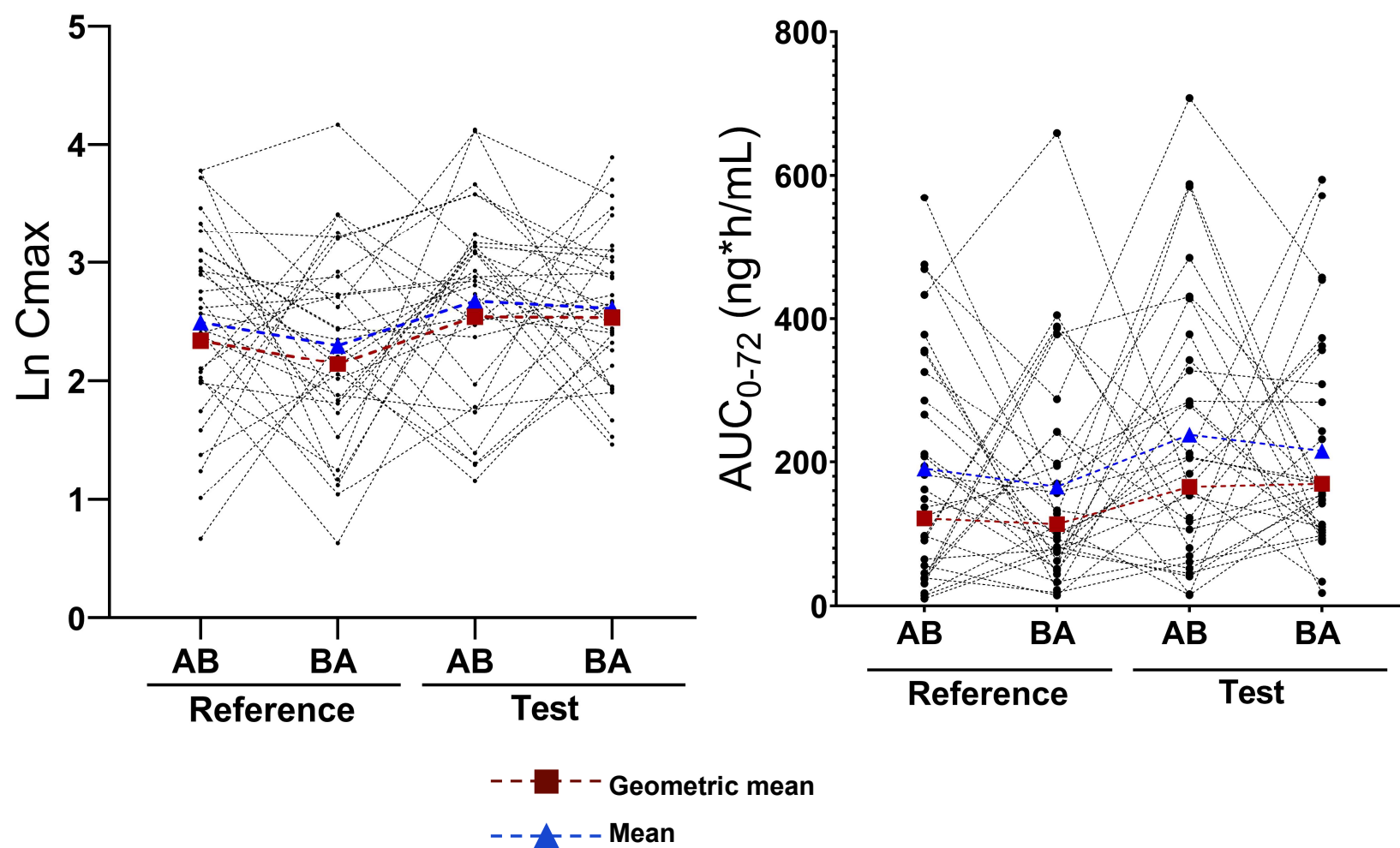

Figure S1. Comparison of individual, mean, and geometric mean of AUC and  $C_{\max}$  by treatment.

Supplement: Supplementary file 1 [file pharmaceuticals-18-01193-s001.zip › pharmaceuticals-3725613_Figure S1.pdf]
